# Supplementary material for: Multiobjective optimization identifies cancer-selective combination therapies
Source: PLoS Comput Biol. 2020 Dec 28;16(12):e1008538. doi: 10.1371/journal.pcbi.1008538 (PMC7793282; doi:10.1371/journal.pcbi.1008538)
Supplement: S1 Text — (PDF) [file pcbi.1008538.s001.pdf]

Determining the parameters of the binary model

As explained in the main text, sigmoidal Hill curves of the form

$$f(\log c, Q_{min}, H, \log IC50) = Q_{min} + \frac{1 - Q_{min}}{1 + (\log c / \log IC50)^H}$$

were fit to measured growth fractions of each monotherapy on all 60 cell lines as a function of logarithmic drug concentration $\log c$. Q_{min} is the bottom asymptote of the growth inhibition curve fitting, $IC50$ is the concentration at which half of the maximal effect is achieved, and H is the so-called Hill coefficient. We used a constrained Hill fit that starts off at 1 and is not allowed to go lower than the second lowest measured value. The lower bound was set because the sensitivity of each drug was measured only in a handful of concentrations, and unconstrained fitting may lead to overestimation of drug efficacies. The lowest growth fraction value was removed from data because it was regarded as an outlier in many cases. The fitting procedure is depicted in Fig. 1 A of the main text.

The interaction term in Eq. [9] of the main text was computed matrix-wise to reduce the effects of plate-to-plate variation in drug screens. Missing drug interaction measurements were imputed with the mean Bliss excess over the cell lines. We also tested an alternative model in which the Bliss excesses were computed against the fitted potencies of single drugs, and in which the regression model would then add up to the actual, measured pairwise therapeutic effects. However, this turned out to yield faulty predictions for combinations of third or higher-order, resulting in a large number negative therapeutic and nonselective effects.
