# Supplementary material for: Multiobjective optimization identifies cancer-selective combination therapies
Source: PLoS Comput Biol. 2020 Dec 28;16(12):e1008538. doi: 10.1371/journal.pcbi.1008538 (PMC7793282; doi:10.1371/journal.pcbi.1008538)
Supplement: S1 Appendix — (PDF) [file pcbi.1008538.s002.pdf]

Nonselective effect and drug toxicity

Nonselective effect computed as the mean effect over all cell lines was used as a surrogate to nonselective toxicity in our multiobjective optimization approach for designing combination therapies. Since all the cell lines in the NCI-60 panel are cancerous, we explored support for the widely-used assumption that the nonselective effect of a drug or a combination of drugs reflects the degree of toxicity on healthy cells as well. To this end, we first divided the 104 drugs in the NCI ALMANAC [1] resource into nine classes according to their mechanism of action (MoA), and investigated the relationship between the nonselective effect and adverse effects in each class separately.

The MoA classes are: alkylating agents, histone deacetylase inhibitors (HDAC inhibitors), hormonal therapies, nucleosides or antimetabolites, platinum therapies, topoisomerase (TOP) targeting and DNA binding compounds, microtubule inhibitors (MTI), drugs that affect cellular signaling, and miscellaneous. The nonselective effects of all monotherapies categorized by their mechanism of action are shown in Fig. S1. From the figure, it can be seen that hormonal therapies and drugs interfering with cellular signaling have low nonselective effect, whereas drugs that bind or damage DNA, inhibit its repair or synthesis, target DNA topoisomerases, histone deacetylases or tubulin have, in general, much greater nonselective effect.

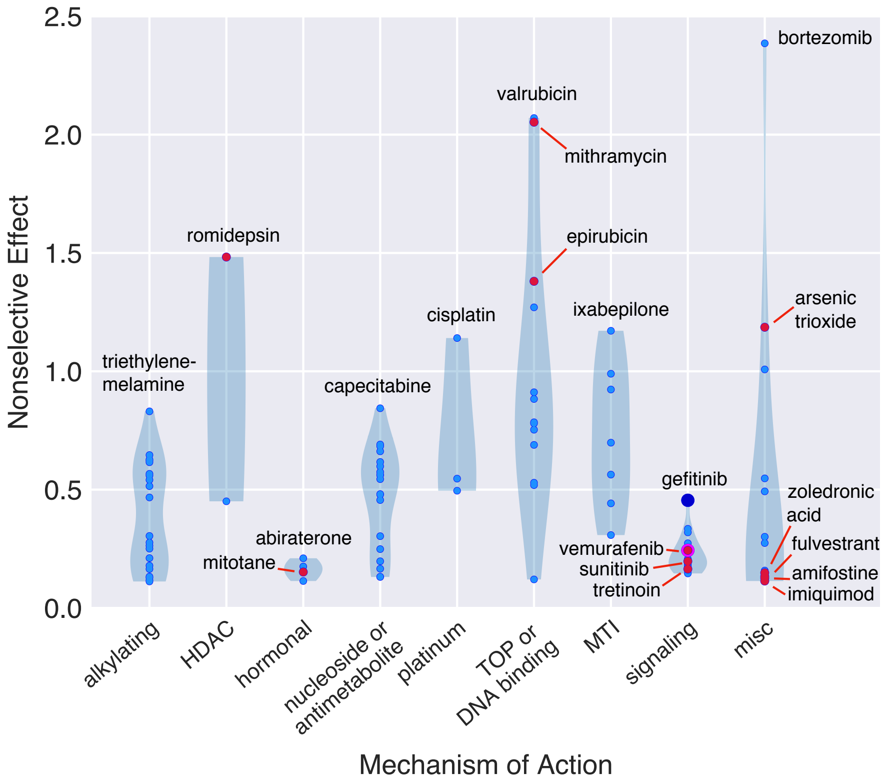

Figure S1: Nonselective effects of monotherapies categorized according to their mechanism of action. Nonselective effect was computed as the mean effect over all the cancer cell lines in the NCI-ALMANAC panel as a surrogate to drug toxicity. The drug with highest nonselective effect in each category is named, as well as the Pareto optimal monotherapies for MALME-3M (red dots). Vemurafenib (big magenta dot) and gefitinib (big blue dot) are highlighted as in Fig.1 C of the main text.

Table S1 lists the molecular targets of the Pareto optimal monotherapies, which are also shown in Fig. S1 as red dots. A higher number of targets does not automatically imply higher nonselective effect. Typically, the drugs with highest nonselective effects are specific for a single general target type such as histone deacetylase, tubulin or DNA. The essential difference between those and the 'signaling' class is that the targets of the signaling class (which are sometimes synonymously called 'targeted' drugs) are more cancer-selective, in the sense that their expression or action is different in healthy cells [2]. The drugs with general targets are therefore expected to show higher toxicity as well. Indeed, it is known that all cytotoxic chemotherapy drugs display a range of severe side effects due to their poor selectivity for cancerous tissue over normal tissue [3].

In the following sections, we list main side-effects of typical drugs within each class, and for each MALME-3M Pareto optimal monotherapy separately. The list shows that there is a general tendency for occurrence of severe side-effects with increasing nonselective effect, but exceptions do exist. References are the online resources Drugs.com (https://www.drugs.com) and DrugBank (https://www.drugbank.ca) unless stated otherwise.

Table S1: Molecular targets of Pareto optimal monotherapies for MALME-3M (listed by DrugBank)

Treatment	$\overline{E}_\delta$	E	#targets	Targets
imiquimod	0.11	0.02	2	TLR7, TLR8
amifostine	0.13	0.03	1	ALPPL2
fulvestrant	0.13	0.03	1	ESR1
zoledronic acid	0.15	0.05	3	FDPS, GGPS1, Hydroxylapatite
mitotane	0.15	0.05	5	CYP11B1, FDX1, ESR1, PGR, AR
sunitinib	0.16	0.08	8	PDGFRB, FLT1, KIT, KDR, FLT4, FLT3, CSF1R, PDGFRA
tretinoin	0.20	0.25	18	RXRB, RXRG, RARG, ALDH1A1, GPRC5A, ALDH1A2, RARRES1, RARA, RARB, LCN1, OBP2A, RBP4, PDK4, RXRA, CYP26A1, CYP26B1, CYP26C1, HPGDS
vemurafenib	0.24	1.60	1	BRAF
arsenic trioxide	1.19	1.92	10	IKBKB, TXNRD1, JUN, CCND1, MAPK3, MAPK1, AKT1, CDKN1A, HDAC1, PML
epirubicin	1.38	1.94	2	TOP2A, DNA
romidepsin	1.48	2.08	5	HDAC1 HDAC2, HDAC4, HDAC6, ABCC1
mithramycin	2.05	3.11	1	DNA

$\overline{E}_\delta$: Nonselective effect from the fitted Hill equation
E: Therapeutic effect from the fitted Hill equation

1. Alkylating agents

Alkylating agents are compounds that work by adding an alkyl group to the guanine base of the DNA molecule, preventing the strands of the double helix from linking as they should. This causes breakage of the DNA strands, affecting the ability of the cancer cell to multiply. Eventually, the cancer cell dies. Since the target is DNA, the drugs in this class are toxic to normal cells, especially those dividing fast, and the drugs have severe side-effects as well. Curiously, the distribution of nonselective effect in this class has a bimodal structure, with one group showing a high nonselective effect, while it is very low in the other group, of the same magnitude as in the 'signalling' class. However, none of the drugs are Pareto optimal for MALME-3M, indicating that also the therapeutic effect was small for the drugs with small nonselective effect.

2. Histone deacetylase (HDAC) inhibitors

Vorinostat and romidepsin are both histone deacetylase inhibitors approved for cutaneous T-cell lymphoma. HDAC inhibitors work by inducing cancer cell cycle arrest, differentiation and cell death. They also reduce angiogenesis and modulate immune response. The most common toxicities of HDAC inhibitors are thrombocytopenia, neutropenia, diarrhea, nausea, vomiting and fatigue. The class exhibits relative specificity against cancerous cells probably due to lack of multiple epigenetic regulatory mechanisms still present in healthy cells [4].

Romidepsin is MALME-3M Pareto optimal with a high nonselective effect in our multiobjective method. Its most commonly reported adverse events include neutropenia, lymphopenia, thrombocytopenia, infections, nausea, fatigue, vomiting, anorexia, anemia, and ECG T-wave changes. The most commonly reported events leading to discontinuation included infection, fatigue, dyspnea, QT prolongation, and hypomagnesemia.

3. Hormonal therapies

NCI ALMANAC contains four hormonal therapies:

1. abiraterone, a derivative of steroidal progesterone used in combination with prednisone for the treatment of metastatic, castration-resistant prostate cancer;
2. megestrol, a progestational hormone used in the palliative treatment of breast cancer;
3. mitotane, an adrenal inhibitor;

4. tamoxifen, a non-steroidal antiestrogen used to treat estrogen receptor positive breast cancers or reduce incidence. It induces apoptosis in estrogen receptor positive cells.

As the drugs within this class function by different mechanisms and have differing indications, also their side-effects vary. They are typically not severe (although acute liver failure has been reported for abiraterone). Commonly reported side effects of abiraterone include decreased serum potassium, increased serum aspartate aminotransferase, increased serum triglycerides, fluid retention, and hypokalemia. The common side effects tamoxifen are amenorrhea, fluid retention, hot flash, nausea, vaginal discharge, vaginal hemorrhage, weight loss, and skin changes. Likewise, the common side effects of megestrol are weight gain, nausea, vomiting, hypertension, vaginal bleeding and discharge, hyperglycemia, asthenia, and rash.

Of hormonal therapies, **mitotane** is Pareto optimal for MALME-3M in our approach. Its common side effects include lethargy and vertigo, gastrointestinal disturbances, elevated liver enzymes, increased plasma cholesterol/hypercholesterolemia, and increased plasma triglycerides/hypertriglyceridemia.

4. Nucleosides and antimetabolites

Antimetabolites (including nucleoside analogs) are chemoteherapy drugs that interfere with one or more enzymes or their reactions that are necessary for DNA synthesis. They affect DNA synthesis by acting as a substitute to the actual metabolites that would be used in the normal metabolism. This leads to the inhibition of DNA and RNA synthesis and cell death. Therefore they are expected to have a range of severe side-effects. Similar to the distribution of alkylating agents' nonselective effect, antimetabolite nonselective effect has high variation, yet the mean value is closer to the mode with higher nonselective effect.

As an example, the drug with the highest nonselective effect in this group is capecitabine, a prodrug, that is enzymatically converted to fluorouracil (antimetabolite) in the tumor, where it inhibits DNA synthesis and slows growth of tumor tissue. Its most commonly reported adverse reactions are gastrointestinal disorders (especially diarrhea, nausea, vomiting, abdominal pain, and stomatitis), hand-foot syndrome (palmar-plantar erythrodysesthesia), fatigue, asthenia, anorexia, cardiotoxicity, increased renal dysfunction in those with preexisting compromised renal function, and thrombosis/embolism.

5. Platinum therapies

The three platinum drugs, carboplatin, cisplatin and oxaliplatin, are effective chemotherapies, but their therapeutic index remain severely limited by the number, and the severity, of their side effects [3]. The side effects experienced by patients treated with a platinum-based chemotherapy drug are similar, although the specific dose-limiting toxicity is different for each drug. For carboplatin the dose-limiting toxicity is myelosuppression, for cisplatin it is nephrotoxicity, and for oxaliplatin it is neurotoxicity. All three platinum therapies also have a significant nonselective effect on the NCI-60 cell lines, and cisplatin's nonselective effect is among the highest 10% in the whole dataset.

6. Topoisomerase targeting and DNA binding compounds

This is a wide class of drugs that typically inhibit DNA and RNA synthesis by interacting with topoisomerases and DNA. It includes anthracyclines, which are among the most important antitumor drugs available. Most anthracyclines have been isolated from natural sources and antibiotics. However, they lack the specificity of the antimicrobial antibiotics and thus produce significant toxicity.

An example of anthracyclines is **epirubicin**, which is MALME-3M Pareto optimal. Epirubicin has antimitotic and cytotoxic activity. It inhibits nucleic acid (DNA and RNA) and protein synthesis through a number of mechanisms of action. In addition to typical side effects (*e.g.* leukopenia, neutropenia, nausea, infections, alopecia) associated with chemotherapy treatments, some severe, life-threatening side effects, such as myocardial damage and heart failure, are reported common for epirubicin. The drug has high nonselective effect on the NCI-60.

Mithramycin (also known as plicamycin) has the highest nonselective effect of all our MALME-3M Pareto optimal monotherapies. It is a DNA/RNA polymerase inhibitor, DNA-binding transcriptional inhibitor, antibiotic, and observed to facilitate tumor necrosis factor (TNF)-α and Fas ligand-induced apoptosis. It has been used to treat testicular cancer, Paget's disease of bone, and chronic myeloid leukemia. The manufacturer discontinued the drug in 2000. It is toxic to bone marrow, liver, and kidneys. It is also lethal to Hela cells in 48 hours at concentrations as low as 0.5 micrograms per milliliter of tissue culture medium. Major side effects are thrombocytopenia and hemorrhagic diathesis that can be life threatening [5]

6. Microtubule inhibitors

Microtubules are important cellular targets for anticancer therapy because of their key role in mitosis. Microtubule inhibitors (MTI) such as taxanes, vinca alkaloids, and epothilones stabilize or destabilize microtubules, thereby suppressing microtubule dynamics required for proper mitotic function, effectively blocking cell cycle progression and resulting in apoptosis [6]. MTIs consist of two important subclasses: mictotubule stabilizing taxanes (cabazitaxel, docetaxel, and paclitaxel) and microtubule destabilizing vinca alkaloids (vinblastine, vincristine, and vinorelbine).

For taxanes, commonly reported side effects of cabazitaxel include neutropenia, diarrhea, nausea, and vomiting. Neutropenic deaths have been reported. Severe neutropenia is also one of the side effect of docetaxel, for which hematologic toxicity is also increased at higher doses and in patients with elevated baseline liver function tests. Severe hypersensitivity reactions, including anaphylaxis have been reported in patients receiving docetaxel or paclitaxel.

All vinca alkaloids make a characteristic peripheral neurotoxicity, although severe neurotoxicity is observed less frequently with vinblastine and vinorelbine as compared with vincristine. Neutropenia is the main dose-limiting toxicity of vinblastine and vinorelbine. Gastrointestinal toxicities, aside from those caused by autonomic dysfunction, may be also observed with using vinca alkaloids [7].

While taxanes and vinca alkaloids all show considerable nonselective effect, ixabepilone, which is not part of the taxane nor the vinca alkaloid family, has the highest nonselective effect in the NCI ALMANAC. However, the main treatment-related adverse events associated with ixabepilone therapy are similar to those reported with other MTIs. The treatment-limiting toxicity is grade 3-4 neuropathy. Ixabepilone-induced neuropathy is primarily sensory and reversible after dose reductions. The most common hematologic adverse events include grade 3-4 neutropenia and leucopenia. Other adverse events include fatigue, arthralgia, myalgia, and diarrhea [6].

8. Cellular signaling

This class consists of small-molecule inhibitors ('nibs'), in particular protein kinase inhibitors, and a few other molecularly targeted compounds. Some proteins that are essential to cellular signaling are often overexpressed in cancers, triggering signaling cascades that lead, for example, to increased survival of cancer cells and uncontrolled cell proliferation. Protein kinase inhibitors work by inhibiting the overexpressed proteins' kinases, thereby inhibiting the downstream signaling cascades as well, which may help stopping malignant cell proliferation. Since the protein targets are overexpressed, they are more cancer cell selective compared to chemotherapeutic targets. Therefore, the kinase inhibitors (and the whole 'signaling' class) exhibits high variation between the therapeutic effect and the nonselective effect, the latter being typically smaller than the nonselective effect in various types of chemotherapy. Also, kinase inhibitors are in general less toxic and, in the right patient population, more potent than conventional chemotherapy [8]. However, like in conventional chemotherapy, development of resistance and unwanted side effects are limitations to kinase inhibitors.

There are three Pareto optimal monotherapies for MALME-3M in this class:

Tretinoin is a naturally occurring derivative of vitamin A indicated for the induction of remission in patients with acute promyelocytic leukemia, for the topical treatment of acne vulgaris, flat warts and other skin conditions. The drug could be equally well in the 'miscellaneous' class because of its special mechanism of action of targeting retinoic acid receptors. The most frequent undesirable effects of this drug are consistent with signs of hypervitaminosis A syndrome. Acute promyelocytic leukemia patients treated with tretinoin may experience a potentially fatal syndrome characterized by fever, dyspnea, acute respiratory distress, weight gain, radiographic pulmonary infiltrates, pleural and pericardial effusions, edema, and hepatic, renal, and multi-organ failure. This syndrome is sometimes accompanied by impaired myocardial contractility and episodic hypotension with or without concomitant leukocytosis.

Sunitinib is multi-targeted receptor tyrosine kinase inhibitor indicated for the treatment of advanced renal cell carcinoma as well as the treatment of gastrointestinal stromal tumor after disease progression or on intolerance to imatinib mesylate. It exhibits antitumor and antiangiogenic properties by inhibiting multiple receptor tyrosine kinases, including platelet-derived growth factors), vascular endothelial growth factors, FMS-like tyrosine kinase-3, colony-stimulating factor type 1, and glial cell-line-derived neurotrophic factor receptor. Commonly reported side effects of sunitinib include oral candidiasis, asthenia, decreased left ventricular ejection fraction, diarrhea, hypokalemia, lymphocytopenia, mucositis, neutropenia, vomiting, and hypertension. Sunitinib has a boxed warning for hepatotoxicity.

Vemurafenib Vemurafenib is a kinase inhibitor with activity against BRAF kinase with mutations, in particular V600E, and it is approved for the treatment of metastatic melanoma with this mutation. In particular, vemurafenib does not have antitumour effects against melanoma cell lines with the wild-type BRAF mutation. Vemurafenib is well tolerated, with the most common adverse effects including skin reactions, photosensitivity, headache, and arthralgia [9].

Biggest problems with vemurafenib therapy are rapid emergence of drug resistance and development of squamous cell carcinoma, the latter of which appears in the few toxicity reports as well.

Gefitinib is an inhibitor of the epidermal growth factor receptor (EGFR) tyrosine kinase. The protein is overexpressed in certain human carcinoma cells, such as lung and breast cancer cells. Geifitinib is not MALME-3M Pareto optimal, but is included here because of somewhat higher nonselective effect in comparison to other compounds in the 'signaling' class (but still on the lower limits for widely toxic classes of chemotherapy). EGFR is the only protein target listed by DrugBank, yet gefitinib is known to have many off-targets [10], which could be one reason for the observed nonselective effect. The other is the location of EGFR protein high in the EGF/EGFR signaling pathway cascade, which is one of the most important pathways in mammalian cells regulating a series of important events including proliferation, migration, differentiation, and apoptosis. The acute toxicity of gefitinib up to 500 mg in clinical studies has been low. In non-clinical studies, a single dose of 12,000 mg/m2 (about 80 times the recommended clinical dose on a mg/m2 basis) was lethal to rats.

9. Miscellaneous

Four of our Pareto optimal drugs cannot be associated with any of the eight categories above. These all show a very small therapeutic on MALME-3 as well as nonselective effect on the NCI-60 cell lines. In fact, the therapeutic effect is smaller than the nonselective effect for all of them, so they are unlikely options for an efficient monotherapy. Also their adverse and toxic effects listed in DrugBank and Drugs.com are usually not severe:

Imiquimod is an immune response modifier that acts as a toll-like receptor 7 agonist. It is commonly used topically to treat warts on the skin of the genital and anal areas. The most frequently reported side effects are local skin reactions, headache, application site reactions, influenza-like illness, fatigue, nausea, and fever.

Amifostine is an organic thiophosphate cytoprotective agent indicated to reduce the cumulative renal toxicity associated with repeated administration of cisplatin in patients with advanced ovarian cancer or non-small cell lung cancer and also to reduce the incidence of moderate to severe xerostomia in patients undergoing post-operative radiation treatment for head and neck cancer. Its common side-effects are hypotension, nausea and hypocalcemia.

Fulvestrant competitively downregulates estrogen receptors, and also degrades them. Both of these mechanisms inhibit the growth of tamoxifen-resistant as well as estrogen-sensitive human breast cancer cell lines. Common side-effects include neutropenia, leukopenia, anemia, thrombocytopenia, vasodilation, elevated hepatic enzyme levels, gastrointestinal problems such as nausea and pain, anorexia, and infections.

Zoledronic acid is a third generation, nitrogen containing bisphosphonate that inhibits osteoclast function and prevents bone resorption. It is indicated to treat hypercalcemia of malignancy, multiple myeloma, bone metastases from solid tumors, osteoporosis in men and postmenopausal women, glucocorticoid induced osteoporosis, and Paget's disease of bone in men and women. It has wide therapeutic window. Fever is the most common adverse effect associated with zoledronic acid infusion. Flu-like syndromes including fever, chills, bone pain, and/or arthralgias and myalgias have also occasionally been reported. These symptoms generally did not require treatment and resolved within 24 to 48 hours. Renal toxicity is the most sever of its common side-effects.

Bortezomib is an interesting drug in the miscellaneous class. It is not Pareto optimal for MALME-3M, and, in fact, it has the highest nonselective effect of all monotherapies in the NCI ALMANAC. Therefore, one would expect it to be highly toxic, and an unlikely candidate for anticancer monotherapy. Quite surprisingly, however, bortezomib is the the first drug that was approved as an anticancer therapy based on NCI-60, and the first proteasome inhibitor anticancer drug [11]. It is indicated for treatment of multiple myeloma and mantle cell lymphoma. Major factors in its discovery were its NCI-60 promiscuity and COMPARE-negativity (meaning its mechanism of action was different from the other therapies: It inhibits the 26S proteasome, which is a protein complex that degrades ubiquitinated proteins in the ubiquitin-proteasome pathway). Also surprisingly, Bortezomib is quite well tolerated and can be administered in the outpatient setting with manageable toxicities. However, it does have severe side effects: Peripheral neuropathy is the most common dose limiting toxicity [12].

Myeloma cell lines or patient-derived myeloma cells have been reported to be at least 170-fold more sensitive to bortezomib compared with peripheral blood mononuclear cells from normal volunteers [13], which indicates that by inhibition of proteasome activity, bortezomib effectively kills cancer cells, while normal cells are affected to a lesser extent [11]. In this sense, bortezomib is perhaps 'the exception that proves the rule': Nonselective effect over NCI-60 usually reflects the toxicity of normal cells, but not always, and therefore nearly Pareto optimal therapies should be investigated as well.

References

[1] Holbeck SL, Camalier R, Crowell JA, Govindharajulu JP, Hollingshead M, Anderson LW, et al. The National Cancer Institute ALMANAC: A Comprehensive Screening Resource for the Detection of Anticancer Drug Pairs with Enhanced Therapeutic Activity. Cancer Res. 2017;77(13):3564–3576.

[2] Blagosklonny MV. Analysis of FDA Approved Anticancer Drugs Reveals the Future of Cancer Therapy. Cell Cycle. 2004;3(8):1033–1040. doi:10.4161/cc.3.8.1023.

[3] Oun R, Moussa YE, Wheate NJ. The side effects of platinum-based chemotherapy drugs: a review for chemists. Dalton Trans. 2018;47:6645–6653. doi:10.1039/C8DT00838H.

[4] Eckschlager T, Plch J, Stiborova M, Hrabeta J. Histone Deacetylase Inhibitors as Anticancer Drugs. International journal of molecular sciences. 2017;18(7):1414. doi:10.3390/ijms18071414.

[5] Holstege CP. Mithramycin. In: Wexler P, editor. Encyclopedia of Toxicology (Third Edition). third edition ed. Oxford: Academic Press; 2014. p. 347 – 348. Available from: `http://www.sciencedirect.com/science/article/pii/B9780123864543007521`.

[6] Perez EA. Microtubule inhibitors: Differentiating tubulin-inhibiting agents based on mechanisms of action, clinical activity, and resistance. Molecular Cancer Therapeutics. 2009;8(8):2086–2095. doi:10.1158/1535-7163.MCT-09-0366.

[7] Moudi M, Go R, Yien CYS, Nazre M. Vinca alkaloids. International journal of preventive medicine. 2013;4(11):1231–1235.

[8] Kannaiyan R, Mahadevan D. A comprehensive review of protein kinase inhibitors for cancer therapy. Expert review of anticancer therapy. 2018;18(12):1249–1270. doi:10.1080/14737140.2018.1527688.

[9] Hagen B, Trinh VA. Managing Side Effects of Vemurafenib Therapy for Advanced Melanoma. Journal of the advanced practitioner in oncology. 2014;5(6):400–410.

[10] Brehmer D, Greff Z, Godl K, Blencke S, Kurtenbach A, Weber M, et al. Cellular Targets of Gefitinib. Cancer Research. 2005;65(2):379–382.

[11] Chen D, Frezza M, Schmitt S, Kanwar J, Dou QP. Bortezomib as the first proteasome inhibitor anticancer drug: current status and future perspectives. Current cancer drug targets. 2011;11(3):239–253. doi:10.2174/156800911794519752.

[12] Field-Smith A, Morgan GJ, Davies FE. Bortezomib (Velcadetrade mark) in the Treatment of Multiple Myeloma. Therapeutics and clinical risk management. 2006;2(3):271–279. doi:10.2147/tcrm.2006.2.3.271.

[13] Hideshima T, Richardson P, Chauhan D, Palombella VJ, Elliott PJ, Adams J, et al. The Proteasome Inhibitor PS-341 Inhibits Growth, Induces Apoptosis, and Overcomes Drug Resistance in Human Multiple Myeloma Cells. Cancer Research. 2001;61(7):3071–3076.
