# Supplementary figures and images for: Multiobjective optimization identifies cancer-selective combination therapies
Source: PLoS Comput Biol. 2020 Dec 28;16(12):e1008538. doi: 10.1371/journal.pcbi.1008538 (PMC7793282; doi:10.1371/journal.pcbi.1008538)

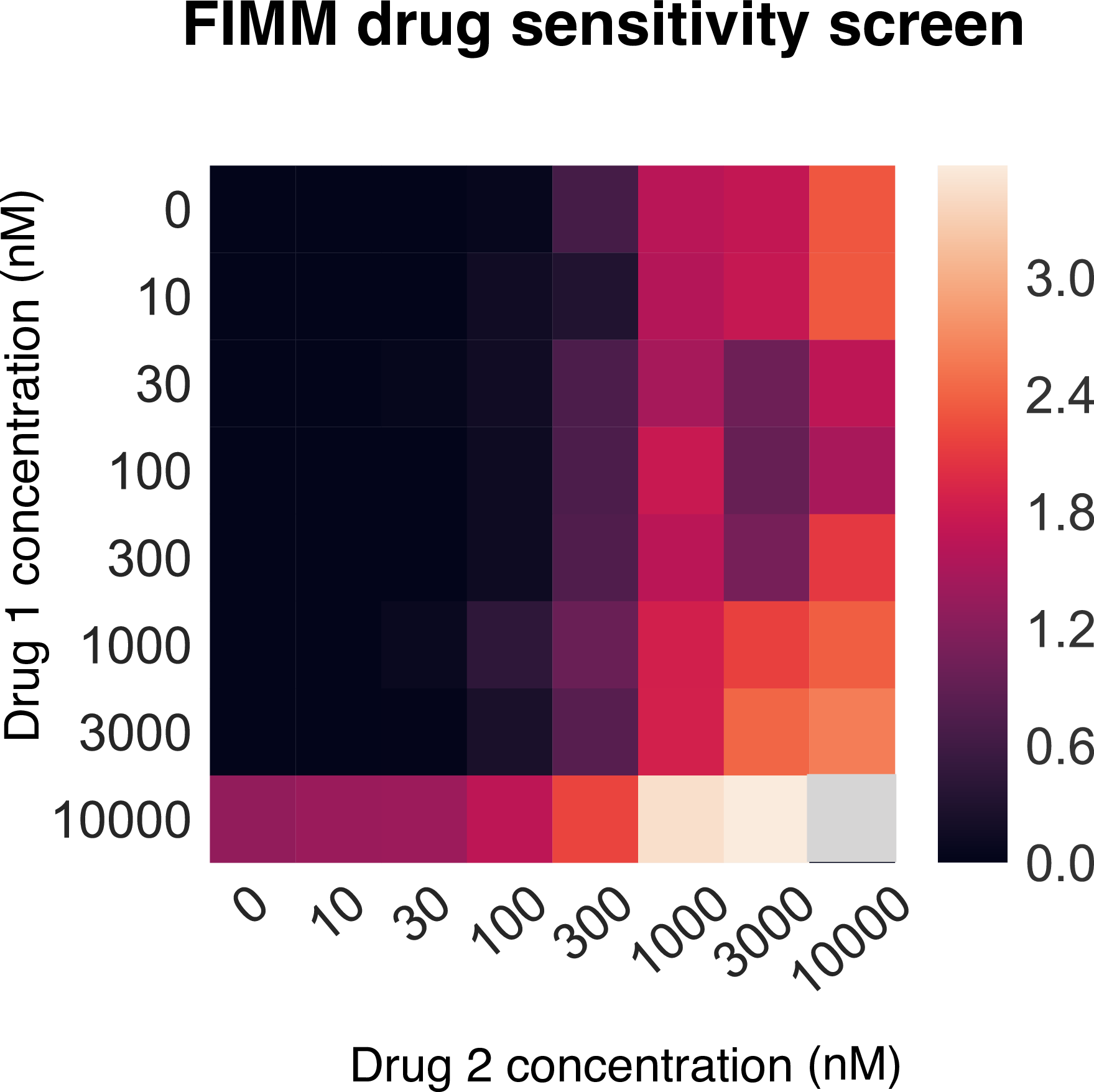

Supplement: S1 Fig — 8-by-8 matrices were constructed from Cell Titer-Glo readouts at concentrations 0 nM, 10 nM, 30 nM, 100 nM, 300 nM, 1000 nM, 3000 nM and 10000 nM of each drug compound (vemurafenib and gefitinib in this example). The plotted quantity is the effect E = −log(Q). (TIF) [file pcbi.1008538.s003.tif]

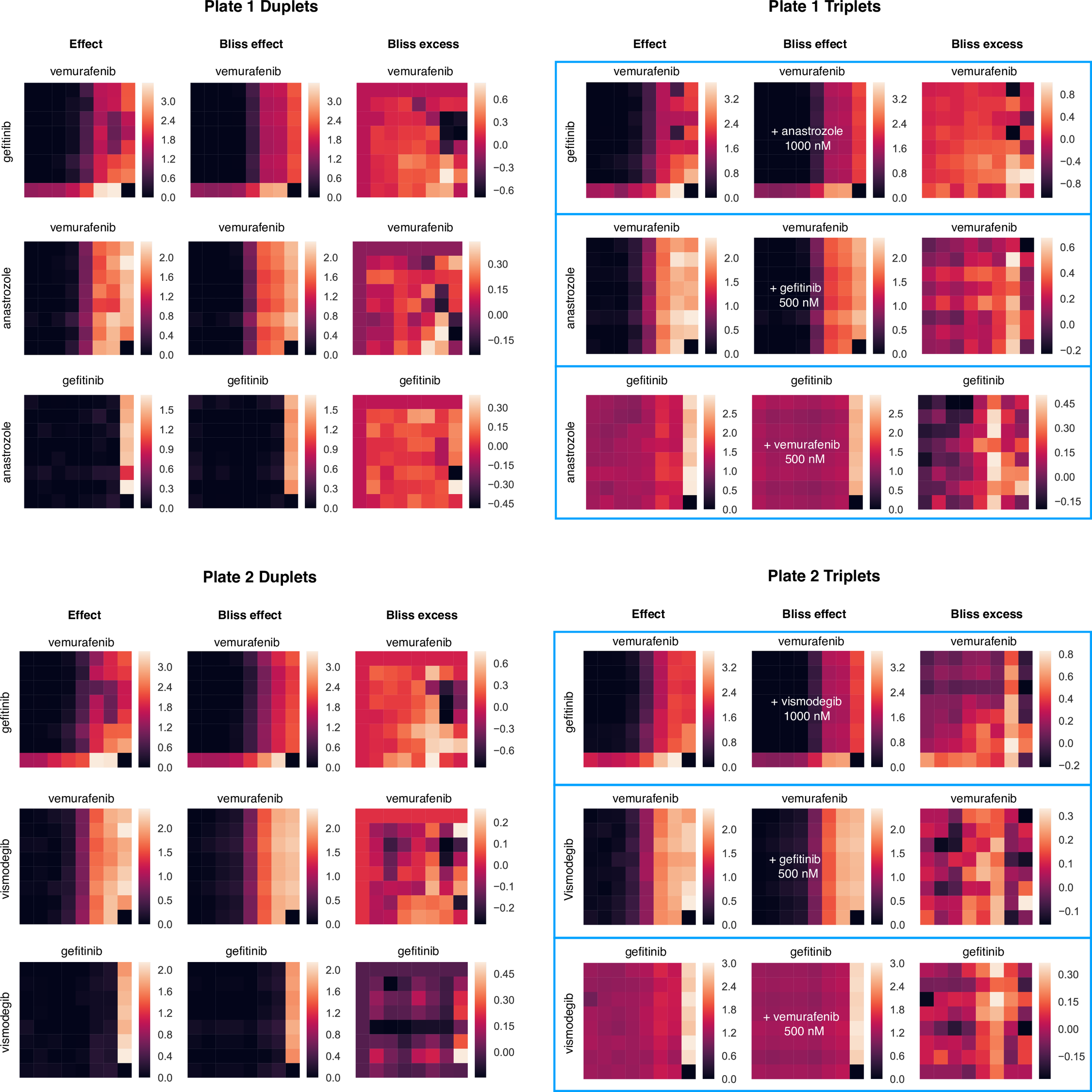

Supplement: S2 Fig — In the in-house combination screen, seven different concentrations in log3-fold dilution ranging from 10 nM to 10000 nM of vemurafenib and gefitinib, vemurafenib and anastrozole, and gefitinib and anastrozole were combined with each other in 8x8 matrix formats in duplicate. For the triple combination, 500 nM of vemurafenib or 500 nM gefitinib or 1000 nM of anastrozole was added to the alternate drug combination matrix (without replicates). In the same way, another set of combination was prepared for vemurafenib, gefitinib and vismodegib. For triple combination, 1000 nM of vismodegib was used whereas the concentrations of vemurafenib and gefitinib were the same as mentioned above. (TIF) [file pcbi.1008538.s004.tif]
