# Supplementary material for: Multiobjective optimization identifies cancer-selective combination therapies
Source: PLoS Comput Biol. 2020 Dec 28;16(12):e1008538. doi: 10.1371/journal.pcbi.1008538 (PMC7793282; doi:10.1371/journal.pcbi.1008538)
Supplement: S1 Table — (PDF) [file pcbi.1008538.s005.pdf]

Treatment	Nonselective effect $\overline{E}_\delta$	Therapeutic effect E
imiquimod	0.11	0.02
amifostine	0.13	0.03
fulvestrant	0.13	0.03
zoledronic acid	0.15	0.05
mitotane	0.15	0.05
sunitinib	0.16	0.08
tretinoin	0.20	0.25
vemurafenib	0.24	1.60
methoxsalen + vemurafenib	0.39	1.74
anastrozole + vemurafenib	0.40	1.90
thalidomide + vemurafenib	0.42	1.93
tretinoin + vemurafenib	0.48	2.08
sirolimus + vemurafenib	0.62	2.44
quinacrine + vemurafenib	0.78	2.45
gefitinib + vemurafenib	0.82	2.99
arsenic trioxide + dactinomycin	1.42	3.04
cisplatin + vemurafenib	1.42	3.08
arsenic trioxide + vemurafenib	1.48	3.43
cisplatin + everolimus	1.54	4.05
arsenic trioxide + quinacrine	1.86	4.15
dactinomycin + tamoxifen	2.23	4.21
everolimus + mithramycin	2.59	4.23
bortezomib + vemurafenib	2.69	4.41
mithramycin + sirolimus	2.75	4.98
mithramycin + vemurafenib	2.75	5.80
mithramycin + valrubicin	4.82	7.85
