# Supplementary material for: Multiobjective optimization identifies cancer-selective combination therapies
Source: PLoS Comput Biol. 2020 Dec 28;16(12):e1008538. doi: 10.1371/journal.pcbi.1008538 (PMC7793282; doi:10.1371/journal.pcbi.1008538)
Supplement: S2 Table — (PDF) [file pcbi.1008538.s006.pdf]

Treatment	Nonselective effect $\overline{E}_\delta$	Therapeutic effect E
imiquimod	0.11	0.02
amifostine	0.13	0.03
fulvestrant	0.13	0.03
zoledronic acid	0.15	0.05
mitotane	0.15	0.05
sunitinib	0.16	0.08
tretinoin	0.20	0.25
vemurafenib	0.24	1.60
methoxsalen + vemurafenib	0.39	1.74
anastrozole + vemurafenib	0.40	1.90
thalidomide + vemurafenib	0.42	1.93
tretinoin + vemurafenib	0.48	2.08
methoxsalen + thalidomide + vemurafenib	0.58	2.10
anastrozole + letrozole + vemurafenib	0.59	2.13
anastrozole + thalidomide + vemurafenib	0.59	2.23
fulvestrant + thalidomide + vemurafenib	0.61	2.24
sirolimus + vemurafenib	0.62	2.44
anastrozole + everolimus + vemurafenib	0.75	2.50
cyclophosphamide + sirolimus + vemurafenib	0.77	2.50
methoxsalen + sirolimus + vemurafenib	0.77	2.56
anastrozole + sirolimus + vemurafenib	0.80	2.76
gefitinib + vemurafenib	0.82	2.99
celecoxib + sirolimus + vemurafenib	0.97	3.02
gefitinib + imiquimod + vemurafenib	0.98	3.13
gefitinib + methoxsalen + vemurafenib	1.00	3.39
anastrozole + gefitinib + vemurafenib	1.03	3.72
gefitinib + tretinoin + vemurafenib	1.11	3.83
gefitinib + vemurafenib + vismodegib	1.21	3.96
gefitinib + sirolimus + vemurafenib	1.24	4.42
gefitinib + quinacrine + vemurafenib	1.68	4.75
cisplatin + everolimus + vemurafenib	1.86	5.46
cisplatin + sirolimus + vemurafenib	1.93	5.55
cisplatin + everolimus + gefitinib	2.04	5.84
cisplatin + everolimus + sirolimus	2.08	5.96
cisplatin + doxorubicin + everolimus	2.12	6.09
arsenic trioxide + quinacrine + vemurafenib	2.15	6.25
dactinomycin + tamoxifen + vemurafenib	3.02	6.62
arsenic trioxide + dactinomycin + tamoxifen	3.10	7.10
clofarabine + mithramycin + vemurafenib	3.46	7.11
mithramycin + sirolimus + vemurafenib	3.50	8.08
gefitinib + mithramycin + vemurafenib	4.18	8.52
gefitinib + mithramycin + vismodegib	4.41	8.67
mithramycin + pentostatin + valrubicin	4.94	10.13
mithramycin + pralatrexate + valrubicin	5.60	10.32
mithramycin + valrubicin + vemurafenib	5.62	10.71
mithramycin + sirolimus + valrubicin	5.64	10.86
doxorubicin + mithramycin + valrubicin	5.77	11.48
