# Supplementary material for: Multiobjective optimization identifies cancer-selective combination therapies
Source: PLoS Comput Biol. 2020 Dec 28;16(12):e1008538. doi: 10.1371/journal.pcbi.1008538 (PMC7793282; doi:10.1371/journal.pcbi.1008538)
Supplement: Attachment — Submitted filename: response_to_reviewers.pdf [file pcbi.1008538.s008.pdf]

Point-by-point replies to comments by the reviewers

Response to Reviewer 1

> **Reviewer #1:** Drug combination is critical for overcoming drug resistance, especially in cancer treatment. In this study, the authors proposed to predict drug combination for specific cancer cells using the multi-objective optimization method. It is a good exploratory design. Some major comments are:

We thank the Reviewer for the expert comments, and apologize that the Reviewer found the methodology presented in the original manuscript somewhat unclear. We have made a number of clarifications in the revised version to make the modeling of combination efficacy more transparent. Please find our point-by-point responses below (page numbers refer to the revised manuscript with changes highlighted):

1. *Equation (3) have no biological meaning, and would be wrong for most drug combinations*

We have chosen to work with the negative logarithm of growth fraction because the therapeutic effect defined this way is additive for (statistically) non-interacting drugs (this fact is discussed in detail *e.g.* in Ref [17]). The same analysis could be carried out with the growth inhibition variables as well but the results would be harder to interpret and to visualize because the therapeutic effect of the Pareto optimal combinations would not then be related in an approximately linear fashion as a function of the nonselective effect, *cf.* Figs 2 A and B. The use of logarithmic scale does not only allow better visualizations but it is also less prone to numerical errors. We also appreciate that the therapeutic effect of drug triplets and higher-order combinations are not exactly given by Eq. (3), but this is a valid and commonly used predictive model for their efficacy based on paired combination data (*e.g.* Refs [21, 22]).

We have now clarified these points better in the text, where the equations for the therapeutic effect are written out separately for monotherapies, two-drug combinations, and higher order combinations (pages 3-4). For instance, Eq. (3) would be an identity for pairwise combinations in the absence of measurement errors. Splitting the effect into monotherapy effects and pairwise Bliss excesses helps to better address the measurement errors because the dataset contains a lot more measurements of monotherapies than of pairwise combinations. This is now described

on page 3 and, as before, on page 6 of the revised manuscript, and in the revised Supporting Information (SI page 6).

2. *Also it is not a regression model*

We are aware of this, but the unfortunate nomenclature is not ours. The model got its name because it was originally used for regression (we have added new reference [23]), inferring interactions between drugs, and then lived on in the literature to mean this particular kind of second order model (see *e.g.* Refs [21, 22]). To avoid confusion, we now call Eq. (3) a 'pair interaction model' instead, and comment in parenthesis that the model is sometimes called 'regression model' (page 3).

3. *Another limitation/concern is the 'pure' data driven, which means a lot of drug combinations should be tested first before the prediction*

The method is indeed designed with high-throughput experimentation in mind. Datasets such as the NCI ALMANAC consisting of nearly 3 million data points allow this kind of analysis. Even if datasets of this size are still rare, and can be produced only with larger resources, the multiobjective optimization method is also applicable to smaller-scale combinatorial datasets that can be produced in many laboratories with the help of robotic instruments. This is now mentioned in the revised Discussion (page 11).

4. *No new knowledge can be mined from this model (considering the biased/ too simple objective function)*

The multiobjective optimization problem at hand has two objective functions: maximizing the therapeutic effect and minimizing nonselective effect of a combination (surrogate for drug toxicity). We note that, to the best of our knowledge, this is the first approach that optimizes both of these objectives simultaneously, whereas most of the previous methods have focused on synergy alone. This multiobjective problem needs to be solved before one can move on to a higher number and/or more complicated objectives, such as maximizing the response of cancerous tissue with intra-tumor heterogeneity *(e.g.* subclonal structure) and tumor microenvironment, while simultaneously minimizing adverse effects of the combinatorial inhibition. The generalization of the method to such cases is now envisaged in the revised Discussion (page 11).

Minors:

5. *Drug i at concentration ci is not correct (for each drug there are multiple doses). The same notation error for Ei(ci,cj, l).*

We have now clarified the definition of the concentration vector ***c*** in the text (pages 3-4). Now it is more clearly pronounced that, in those equations, each drug appears in a combination in one concentration only, not all of them simultaneously. Also, the notation in Eq. (3) has been re-checked.

Response to Reviewer 2

We thank Dr Rampadarath and Dr Nickerson for making the effort of reading carefully through the Jupyter notebooks and for the Reproducibility report.

1. *Although these results can be produced by running this notebook, improved documentation is required to enable independent implementation or reuse of the novel drug-centric mathematical optimization framework developed in this paper*

The notebooks have now been combined and moved to Github (github.com/pulkkinen/combimop), and a README file with detailed implementation instructions has been provided on the repository front page. More comments were added to the notebook itself. We hope that these changes will make independent implementation and reuse easier.

Response to Reviewer 3

> **Reviewer #3:** This manuscript explores how non-selective synergistic drug combinations can be identified for a given cancer cell line using NCI-Almanac data. This is posed as a two-objective optimisation problem aiming at identifying combinations that maximise both therapeutic effect and selectivity for the intended cancer cell line. The study focuses on a BRAFV600E-mutant melanoma cell line (MALME-3M), for which they find several optimal solutions (vemurafenib monotherapy and some of its combinations). Two three-drug combinations (triplets) were tested in vitro and the compromises between their non-selectivity and therapeutic effect discussed.

This is definitely an interesting study. I feel however that it has to be polished before publication.

We thank Dr Ballester for the insightful comments. We found them really helpful in improving the quality and readability of the article. Below please find our point-by-point response to the comments (page numbers refer to the revised manuscript with changes highlighted):

1. *This study employs non-selectivity (essentially which proportion of NCI60 cell lines is inhibited by the drug or combination) as a surrogate of drug safety (toxicity). This assumption is appealing because enables the analysis without requiring further data (e.g. activity of the drug/s on a non-tumoural cell line). Ideally, this plausible hypothesis should be supported by some form of validation.*

We thank the Reviewer for the excellent comment. The revised SI now contains a figure that compares the nonselective effects of drugs in the NCI ALMANAC classified by their mechanism of action: alkylating agents, histone deacetylase inhibitors, hormonal therapies, nucleosides or antimetabolites, platinum therapies, topoisomerase targeting and DNA binding compounds, microtubulin inhibitors, drugs that affect cellular signaling, and miscellaneous drugs. Of these, hormonal therapies and the ‘signalling’ class mostly consisting of targeted inhibitors have systematically lower nonselective effect (the mean over NCI-60 cell lines) than chemotherapies. In particular, platinum therapies and the drugs targeting topisomerase, mictotubulin, or histone deacetylase show much higher nonselective effects. This supports our assumption since the non-targeted therapies, in general, are known to result in more severe side effects. Furthermore, we collected lists of commonly reported and severe adverse effects and toxicities for each of these class, and discussed those identified as MALME-3M Pareto optimal monotherapies in more detail in the revised SI. This qualitative analysis shows that the nonselective effect reflects, in general, the reported nonselective toxicities of drugs, but there are exceptions (*e.g.* bortezomib), and those are discussed as well (pages 1-6 of the revised SI).

2. *Looking at adverse effects arising from clinical practice (drugs.com) and Figure 1C, NCI60-selective vemurafenib has a higher number of important adverse effects than NCI60-promiscuous mythramycin, and thus the assumption does not seem to hold in this case.*

It is true that the list of all side effects of vemurafenib is longer in Drugs.com than of mithramycin, and there are indeed also severe side effects: patients on vemurafenib

often (20 %) develop squamous cell carcinoma. However, this is a very long-term side effect and outside the application scope of this study. Mithramycin instead has direct toxic effects: it causes a life-threatening bleeding syndrome, and it is toxic to bone marrow, liver, and kidneys. Mithramycin is also lethal to Hela cells in 48 hours at concentrations as low as 0.5 micrograms per milliliter of tissue culture medium. Both of these drugs are now discussed in detail in the new SI (SI pages 4-5).

3. *The other aspect to discuss is related to intra-tumour heterogeneity, which is minimal in cancer cell lines (one clone at low passages) but typically high in primary tumours (clones with different drug sensitivies). If we see each melanoma cell line as a tumour clone, it is not unreasonable to think that in vitro non-selective drugs might be able to delay the emergence of acquired resistance more than in vitro selected drugs due to the former neutralising a larger proportion of the tumour clones. In that case, non-selectivity would be positive rather than negative. I think that the study would benefit from discussing the limitations of this assumption.*

This is a very good point, and a common caveat of studies that are based on cancer cell lines such as those from NCI-60. We have added a new paragraph in Discussion pointing out that, *in vivo,* some degree of nonselectivity may be beneficial because multitargeting the tumor subclones and their microenvironments may help thwart multiple mechanisms of tumor proliferation (page 11). However, we think that the complementary objectives of drug efficacy and safety are still valid. The multiobjective method can also be extended to drug profiling data of primary patient samples (*e.g.* blood cancers) or complex 3D models like organoids, co-cultures or even tissue slices, which contain clonal heterogeneity and tumor microenvironmental structure. These generalizations, however, are outside the scope of the present work, and therefore, these aspects are included in Discussion as future work (page 11).

4. *Were all single-drug activities for a given cell line considered in calculating Qmin? If not, how were extreme values discarded?*

Only the lowest measured Q value (growth inhibition percentage) was discarded when fitting the Hill curves to monotherapy dose-response data. Also, the fit was constrained in such a way that the estimated IC50 must be within the range of measurement concentrations, and Qmin could not be lower than the lowest point (after removal of the extreme). Even if there are many measurements for each monotherapy, the number of concentrations is relatively limited, and unconstrained fitting therefore would lead to unrealistic values for IC50 and Qmin. The fitting procedure is explained in the SI (SI page 6).

5. *The authors should discuss how Figure 1C changes depending on the level of variability in single-drug activities.*

We believe that Fig. 1C would not change much if the level of variability in single-drug activities was changed because it is based on the least squares fit of Hill functions based on a quite large number of data points. The interaction terms, on the other hand, may be much more sensitive to measurement errors. We have added a comment on this in the main text (page 3).

6. [*The Theory section*] *should be made clearer and better organised. Adding subsections such as Monotherapy, Two-drug Combinations and Higher-order Combinations would be helpful, each with their specific therapeutic effect and non-selectivity equations.*

We thank the referee for this good suggestion. We have now added subsections with their own equations for the cases m=1, 2, and m > 2.

7. *The two-objective optimization problem is solved for monotherapies and two-drug combinations by inspecting NCI-Almanac data (a reference to the epsilon-constrained method is required). It is not clear either how this is done for the three-drug combinations, as such data is not available at NCI-Almanac*

The predicted therapeutic effect and the consequent nonselective effect of a drug triplet or any higher order combination is indeed computed from the model containing only pairwise interactions. This is now stated more clearly in the text as the different cases are discussed in their own subsections (pages 3-4). References to epsilon-constraint method are now also included.

8. *I am missing the definition of M in equation (6)*

The paragraph defining the multiobjective optimization problem now starts with the definition of M (page 6), where we define *M* as the maximal number of drug components in a combination.

9. *result to discuss: vemurafenib + gefitinib's synergy depends on their concentration to the extent of being antagonistic for some concentration pairs. Could the authors discuss which of these in vitro concentrations would be relevant in vivo?*

The antagonism occurs only at higher concentrations of vemurafenib and lower concentrations of gefitinib in vitro. However, it is rather impossible to know whether the effects would be observable at therapeutic doses also in vivo, due to many factors affecting the in vivo treatment effects not captured by the in vitro models. We refrain from commenting more on this, because the problem would require a separate investigation, but have discussed the differences between in vitro and in vivo models this in the limitations section of the revised discussion (page 11).

10. *I am missing a summary of which two-objective-optimal monotherapies, two-drug combinations and higher-order combinations have been identified in NCI-Almanac and, in the latter case, confirmed in vitro by the authors.*

Selected Pareto optimal monotherapies and combinations (the ones also highlighted in Figs. 2B and 3) are now listed in new Table 1. Complete lists of MALME-3M Pareto optimal combinations are listed in Tables S2, S3, and S4 of the SI. The in-house experimental validations are listed in Table 2 of the main text.
