# Supplementary material for: Multiobjective optimization identifies cancer-selective combination therapies
Source: PLoS Comput Biol. 2020 Dec 28;16(12):e1008538. doi: 10.1371/journal.pcbi.1008538 (PMC7793282; doi:10.1371/journal.pcbi.1008538)
Supplement: Attachment — Submitted filename: response_to_reviewers_rev2.docx [file pcbi.1008538.s009.docx]

**Point-by-point replies to comments by the reviewers**

**______________________________________________________________________________________________________________________________________**

**Response to Reviewer 1**

**Reviewer #1:** The revision adressed the comments, and is acceptable with minor revisions.
Minor comments:
1) it is better to discuss the potential limitations mentioned in the review.
2) It is better to discuss whether the model can predict new combinations (without combination experimental data), or select the 'best' combinations based on the screening data of drug combinations.

We thank the reviewer for the continued comments on our revised manuscript. In the previous revision round, we discussed many of the limitations of the current methodology. As requested, we have now further discussed these potential limitations of the methodology in the revised version. The following paragraph was added in Discussion (highlighted with green colour in the marked-up manuscript):

To select the Pareto optimal multi-drug combinations without relying on approximative modeling of combination efficacies, the current MOP framework requires as input the efficacies of a majority of combinations both in the target cell line and in a healthy control, or in a sufficiently large number of other cell lines, in order to compute the nonselective effects. More specifically, for finding the Pareto optimal drug combinations of at most M components from a set of N drug compounds, an order of N^M of measurements are required for each cell line used in the modeling. Such large-scale drug screens currently exist for monotherapies and two-drug combinations (M = 1, 2), and to apply the MOP framework to higher-order combinations, it must be augmented with a model that predicts the higher-order combination efficacies. In the present work, we chose the pair interaction model that uses only the measured efficacies of monotherapies and two-drug combinations for this purpose, but there are also other alternatives, which may be based on pair combination efficacies [21,22] or use additional chemical and biological information such as quantitative structure-activity relationship analysis of the compounds, gene expression data, or signaling pathway and network annotations [8, 32].

**___________________________________________________________________**

**Response to Reviewer 3**

**Reviewer #3:** The authors have done an excellent job at addressing all my comments and I agree that the paper has improved very much as a result. I do not have any further comment.

We thank Dr Ballester again for helping us to improve the quality and presentation of the article.
